# Divergence in the activity of the N- and C- catalytic domains of ACE1 - implications for the role of the renin-angiotensin system in Alzheimer’s disease

**DOI:** 10.1186/s40478-019-0718-2

**Published:** 2019-04-24

**Authors:** Noura S. AL Mulhim, Patrick G. Kehoe, J. Scott Miners

**Affiliations:** Dementia Research Group, Translational Health Sciences, Bristol Medical School, University of Bristol, Level 1 Learning and Research, Southmead Hospital, Bristol, BS10 5NB UK

**Keywords:** Angiotensin-II converting enzyme-1 (ACE1), Alzheimer’s disease, Renin-angiotensin system, Anti-hypertensives

## Main text

Angiotensin II converting enzyme-1 (ACE1) now has a recognised role in the pathogenesis of Alzheimer’s disease (AD). ACE1 converts angiotensin-I (Ang-I) to angiotensin-II (Ang-II) and is the rate-limiting enzyme of the classical RAS axis that is commonly known for regulating blood pressure. ACE1 is overactive within the brain in AD and is associated with cognitive decline and disease pathology [16] via overproduction of Ang-II (a potent vasoconstrictor) and its downstream effects mediated by angiotensin-II type 1 receptor (AT1R) signalling. The angiotensin hypothesis of AD describes how Ang-II signalling contributes both directly and indirectly to the development of disease pathology in AD [9], which is supported by clinical observational and pharmaco-epidemiological studies indicating that commonly prescribed ACE1 inhibitors (ACE1 Is), used to treat hypertension, lower the incidence and rate of cognitive decline in AD [2, 10, 15] and are associated with reduced Aβ and Tau pathology [5, 6]. Yet, the role of ACE1 in AD is complicated by seemingly paradoxical associations whereby polymorphisms in *ACE1,* associated with *lower* levels of enzyme production (akin to a net result of ACEIs), are risk factors for AD [4, 12, 14]. This divergent role of ACE1 may be partly explained by studies in cell and mouse models of AD showing that ACE1 has both endopeptidase and carboxypeptidase activity and is capable of degrading Aβ in vitro [7, 8, 18, 19] and in vivo [21] although not all studies are supportive [3]. These dual properties of ACE1 seem somewhat contradictory and make understanding the role of ACE1 in AD challenging, particularly as ACE1 activity in brain tissue in AD correlated with, rather than inversely correlated with, measures of Aβ pathology [16].

Somatic ACE1 contains two homologous catalytic domains: the N- and C-domain, which have different substrate specificities. The C-domain is reported to preferentially convert Ang-I to Ang-II [1], whilst the N-domain favours cleavage of amyloid beta (Aβ) at Asp5His6 [19], as well as having carboxypeptidase activity that promotes the conversion of Aβ42 to Aβ40 [20]. We tested the hypothesis that domain-specific changes in ACE1 in AD would favour elevated Ang-II production whilst impeding Aβ degradation. We report novel findings in post mortem AD brain tissue, using novel immunocapture-based enzyme activity assays, that the activity of the two catalytic domains of ACE1 are significantly altered in opposing directions in AD.

We studied brain tissue from 72 AD and 48 controls obtained from South West Dementia brain bank tissue bank, University of Bristol, UK with ethics committee approval. Cohorts were approximately matched for age-at-death (AD Mean = 78.04, SD = 10.41; controls Mean = 79.42, SD = 9.89), post-mortem delay (PM) (AD Mean = 45.86, SD = 25.8; controls Mean = 48.25, SD = 37.96) and gender (AD = 27 M: 45 F; controls = 29 M: 19 F). AD cases were diagnosed according to international neuropathological guidelines [17]. Controls were cognitively normal and had few or absent neurofibrillary tangles, a Braak stage less than 3, and no other neuropathological abnormalities. ACE1 C-domain and N-domain activity was measured by immunocapture-based FRET assays. Mouse monoclonal anti-human ACE (R&D systems, UK) (0.5 mg/ml) was used in both assays to coat 96-well plates (Nunc MaxiSorp), which were blocked in PBS:1% bovine serum albumin (BSA) before tissue homogenates prepared in 1% SDS lysis buffer (5 M NaCl, 1 M Tris pH 7.6) (diluted 1:5) for C domain activity and (diluted 1:17) for N-domain activity, recombinant human ACE1 (500–7.8125 ng/ml) (R&D systems, UK) were added. Fluorogenic activity following ACE1 cleavage was measured by addition of C-domain or N-domain FRET substrates (Abz-LFK (DnP)-OH trifluoroacetate salt) (Sigma-Aldrich, UK) (0.14 mM) and (Abz-SDK (DnP)-P (Enzo Life Sciences, UK)) respectively and measured with excitation at 320 nm and emission at 405 nm in a fluorescent plate reader (FLUOstar OPTIMA, BMG labtech, UK) (0.68 mM) after 24 h incubation at 37 °C. Captopril (10uM) or 10 μl of distilled water was added to inhibited and uninhibited wells respectively and incubated for 10 min at 37 °C prior to the addition of the FRET substrates.

**Fig. 1:**
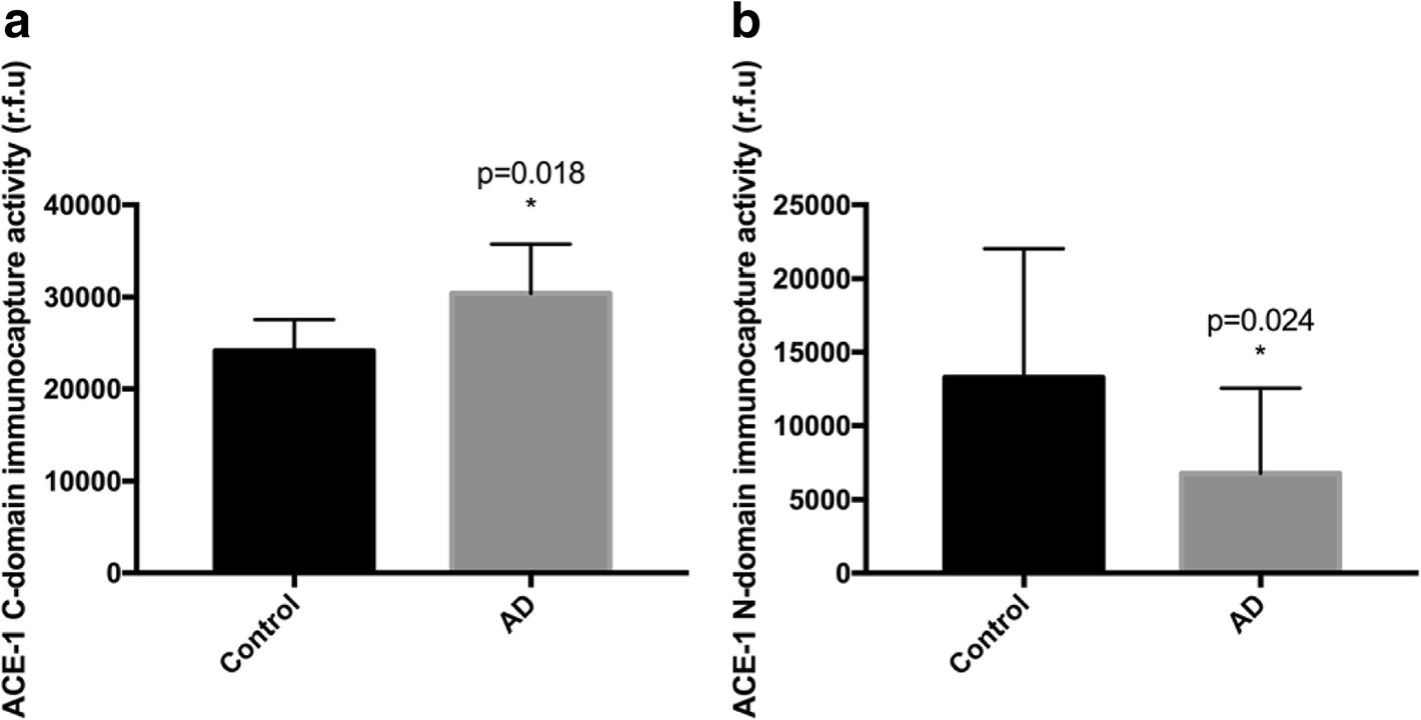
Divergent activity of ACE1 domains in Alzheimer’s disease. Bar charts showing (**a**) significantly higher ACE1 C-domain (Ang-II production) activity in AD compared to age-matched controls and (**b**) significantly reduced ACE1 N-domain activity (Aβ degradation) compared to age-matched controls in the mid-frontal cortex in AD (*n* = 72) and age-matched (*n* = 48). Bars show the median and 95% CI, Mann-Whitney test was used to compare ACE1 C-domain activity between groups. *p* < 0.05 was considered statistically significant

ACE1 C-domain activity was significantly elevated in AD by 25.85% (median = 30,407 rfu in AD compared to median = 24,161 rfu in controls) (*p* = 0.018) (Fig. 1a). In contrast, ACE1 N-domain activity was reduced by 49.18% in AD compared to controls (median = 6750 rfu compared to median = 13,283 rfu in controls) (*p* = 0.024) (Fig. 1b).

Our findings show that ACE1 catalytic domain activity is significantly altered in AD. ACE1 C-domain activity, largely responsible for Ang-II production is significantly increased in AD by ~ 25%, whereas N-domain activity, likely contributing to Aβ cleavage and clearance, is reduced by ~ 50% in AD. These data provide a possible explanation for the divergent role of ACE1 in AD. The combined effect of the domain-specific alterations would favour Ang-II mediated disease progression, likely involving other Ang-II linked AD-related pathological processes according to the Angiotensin hypothesis of AD [11] but also result in impeded Aβ clearance (via reduced N-domain activity) that is predicted to be protective in AD [13]. Our findings may also provide for the first time, a mechanistic explanation for the apparent discrepant findings in previous pharmaco-epidemiological studies and AD risk and progression. Our data points to the need for greater clarity on the extent to which different ACE-Is interact with the two domains on ACE1 and lends credence to the potential value of the development of domain-selective (C-domain) ACE-I’s, that can continue to fulfil their hypertension-treating role, whilst avoiding any potential interference with Aβ clearance and degradation.
